# A reliable and reproducible protocol for sound-evoked vestibular myogenic potentials in rattus norvegicus

**DOI:** 10.3389/fnint.2023.1236642

**Published:** 2023-09-05

**Authors:** Federica M. Raciti, Yasniary Morales, Hillary A. Snapp, Suhrud M. Rajguru

**Affiliations:** ^1^Department of Otolaryngology, University of Miami, Miami, FL, United States; ^2^Department of Biomedical Engineering, University of Miami, Miami, FL, United States; ^3^Bruce W. Carter Department of Veterans Affairs Medical Center, Miami, FL, United States

**Keywords:** cervical vestibular evoked myogenic potential, cVEMP, vestibular system, saccule, vestibular loss, balance, vestibular physiology, animal models

## Abstract

**Introduction:**

Cervical vestibular evoked myogenic potentials (cVEMPs) provide an objective measure of the integrity of the sacculo-collic pathway leading to their widespread use as a clinical tool in the diagnostic vestibular test battery. Though the application of cVEMPs in preclinical models to assess vestibular function, as performed in relevant clinical populations, remains limited. The present study aimed to establish a rodent model of cVEMP with standardized methods and protocols, examine the neural basis of the responses, and characterize and validate important features for interpretation and assessment of vestibular function.

**Methods:**

We compared air-conducted sound (ACS)-evoked VEMPs from the sternocleidomastoid muscles in naïve Brown Norway rats. A custom setup facilitated repeatable and reliable measurements which were carried out at multiple intensities with ACS between 1 and 16 kHz and over 7 days. The myogenic potentials were identified by the presence of a positive (P1)-negative (N1) waveform at 3–5 ms from the stimulus onset. Threshold, amplitude, and latency were compared with intensity- and frequency-matched responses within and between animals.

**Results:**

cVEMP responses were repeatedly evoked with stimulus intensities between 50–100 dB SPL with excellent test-retest reliability and across multiple measurements over 7 days for all frequencies tested. Suprathreshold, cVEMP responses at 90 dB SPL for 6–10 kHz stimuli demonstrated significantly larger amplitudes (*p* < 0.01) and shorter latencies (*p* < 0.001) compared to cVEMP responses for 1–4 kHz stimuli. Latency of cVEMP showed sex-dependent variability, but no significant differences in threshold or amplitude between males and females was observed.

**Discussion:**

The results provide a replicable and reliable setup, test protocol, and comprehensive characterization of cVEMP responses in a preclinical model which can be used in future studies to elucidate pathophysiological characteristics of vestibular dysfunctions or test efficacy of therapeutics.

## 1. Introduction

Housed in the inner ear, the vestibular system detects movement providing critical inputs required for balance and posture. Not only is it essential in maintaining spatial orientation and stabilizing gaze, but the vestibular system has also been associated with memory and cognitive functions (Liu et al., 2004; Zheng et al., 2004, 2006, 2012; Baek et al., 2010). Disorders affecting the vestibular system are most often peripheral in nature, typically affecting one or more vestibular end organs, including the otoliths (Chau et al., 2015; Hulse et al., 2019). Both the utricle and the saccule play an important role as they are primarily responsible for detecting linear acceleration and sensing gravity (Fernandez and Goldberg, 1976). Two of the most common vestibular pathologies, benign paroxysmal positional vertigo (BPPV) and Meniere’s Disease, directly affect otoliths activity (Denholm, 1993; Oku et al., 2003; Zhou et al., 2019), leading to instability, increased fall risk, and reduced quality of life. It has also been shown that these end organs are particularly susceptible to damage arising from hazardous noise or blast exposures (Manabe et al., 1995; Fausti et al., 2009; Lien and Dickman, 2018; Alqudah, 2019; Stewart et al., 2020). Pre-clinical studies provide evidence that noise overexposure often leads to cellular damage in the peripheral vestibular system (Hsu et al., 2008; Akdogan et al., 2009; Tamura et al., 2012) with a well-characterized effect on the response of otolith organs (Sohmer et al., 1999; Biron et al., 2002; Perez et al., 2002; Hsu et al., 2008; Stewart et al., 2018). Clinical studies highlight the functional deficits, increased imbalance and cognitive changes that occur in patients with noise-induced hearing loss (Agrawal et al., 2009; Wu and Young, 2009; Kumar et al., 2010; Akin et al., 2012; Viola et al., 2020). In a companion paper, we ascertained changes induced in the vestibular periphery by noise exposures in a group of firefighters with largely normal clinical audiometric findings and no reports of imbalance or dizziness (Snapp et al., 2023). We observed significant changes in the sacculocollic pathway in firefighters using electrophysiologic measurements when compared to age-matched controls. These subclinical effects of noise exposures on the vestibular system may point to potential hidden vestibular loss. Pre-clinical models characterizing the disease course arising from pathologies of the peripheral vestibular system using clinically validated test battery remain limited, yet are critically important to further our understanding of these findings.

Over the years, numerous diagnostic tests have been developed to provide a comprehensive assessment of the vestibular function in human subjects. As part of the neuro-otological test battery, vestibular evoked myogenic potentials (VEMPs) are a well-established neurophysiological technique used to evaluate the functionality of the vestibular end organs and the integrity of the corresponding neural pathways (Colebatch and Halmagyi, 1992). VEMPs are short-latency alterations of myogenic activity evoked by high-intensity stimuli, either bone-conducted vibration (BCV) or air-conducted stimuli (ACS), and they are typically recorded from the sternocleidomastoid muscle (cervical–cVEMPs) (McCue and Guinan, 1995; Ozeki et al., 1999; Wu and Young, 2002; Young et al., 2002; Colebatch et al., 2016) or the extraocular muscles (ocular–oVEMPs) (Colebatch et al., 1994, 2016; Curthoys et al., 2012). Because of the vestibular projections to the neck and face muscles, VEMPs measurements give valuable information on the status of the vestibulo-collic reflex pathway and either utricular or saccular function specifically (Bolton et al., 1992; Uchino, 1997; Uchino et al., 2005; Uchino and Kushiro, 2011). Since the work by Colebatch et al. (1994), this non-invasive and relatively simple technique has gained significant attention in the field of vestibular diagnostics. VEMPs can be utilized to assess the function of each labyrinth separately, and have been used in characterization of vestibular activity and associated disorders. However, unlike other methods, cVEMP testing offers advantages in terms of patient comfort, technical simplicity, equipment availability, and cost-effectiveness, making it a valuable tool for assessing vestibular function in various clinical settings (Fife et al., 2017).

Common electrophysiological metrics such as threshold, amplitude, and latency, are used clinically to describe VEMP responses. In noise exposed human subjects, previous studies have shown reduced response rate, significant threshold and latency shifts of the myogenic potentials, and changes in VEMP amplitude (Wang and Young, 2007; Kumar et al., 2010; Akin et al., 2012). Although prior work conducted in various mammalian models including mouse (Mikaelian, 1964), rat (Zhu et al., 2011), guinea pig (Murofushi et al., 1995, 2017; Curthoys et al., 2016), cat (Ikegami et al., 1994; McCue and Guinan, 1994; Uchino et al., 2005), chinchilla (McCue and Guinan, 1995), and non-human primates (Young et al., 1977; Corneil et al., 2002; Mitchell et al., 2013) characterized the neural basis of sound- or vibration-evoked myogenic potentials (see Corneil and Camp, 2018 for a comprehensive review), the use of VEMP measurement in preclinical studies to characterize the pathophysiology of vestibular dysfunctions has been rather limited due to the low level of reproducibility and high variability of the responses elicited (Sakakura et al., 2003; Ya et al., 2016). While species-specific morphometric characteristics of the head-neck musculature might partially explain the differences in VEMPs from different animal models (Abrahams and Richmond, 1988; Wilson et al., 1995; Xiang et al., 2008), these challenges are likely associated with the variability in stimulus delivery and recording techniques used. Hence, it is imperative to develop a standardized VEMP test protocol in preclinical models that closely mimics the clinical methodologies. Such a protocol would facilitate robust data collection, interpretation, and comparison between animal studies and human clinical trials.

In this manuscript, we developed and tested a custom set-up and protocol to measure reliable and repeatable sound-evoked cVEMPs in a rat model. We further provide a characterization of various parameters associated with cVEMPs measured in naïve animals that may serve future investigations of vestibulopathies in preclinical models, including but not limited to noise-induced vestibular loss, and therapies for clinical translation.

## 2. Materials and methods

In the present work, cervical myogenic potentials are recorded from the sternocleidomastoid muscles in naïve Brown Norway rats from Charles River Laboratories (strain code 091) and characterized in a cross-sectional study. A subset of this cohort is used to continue to the longitudinal phase of this work. The animals were selected at random while maintaining equal sex and group stratification. In this group, vestibular tests were repeated up to a week after the initial cVEMP measurement to assess within and between subject effects on the long-term reliability of amplitude, latency, and threshold of the cVEMP response in a pre-clinical model. All animal studies were carried out following protocols approved by the University of Miami Institutional Animal Care and Use Committee (IACUC) and followed the recommendations of the Guide for the Care and Use of Laboratory Animals (National Research Council).

### 2.1. Animals

Female (*n* = 4) and male (*n* = 4) Brown Norway rats (14–18 weeks of age) were used in the experiments (Charles River Laboratories, Malvern, PA, USA). We note that the present study aimed to establish relevant methods, protocols, and define characteristics of cVEMPs in response to acoustic stimuli in a preclinical model. Given the scarcity of literature and standardized protocols in this domain, our choices regarding effect sizes and sample size were driven by practical feasibility, challenges associated in working with preclinical models and the pursuit of new insights of these electrophysiological responses. The rats were kept in a climatically controlled environment with a 12-h light/dark cycle conditions and *ad libitum* access to food and water. To perform the functional assessments presented in this work, the rodents were anesthetized with ketamine and xylazine cocktail (44 mg/kg and 5 mg/kg) administered via intramuscular injections with sterile saline solution. The depth of anesthesia was assessed every 15 min with a paw reflex and maintenance doses were given when necessary. The core body temperature was maintained between 37–38°C throughout the procedures.

### 2.2. Vestibular testing (cVEMP)

**FIGURE 1:**
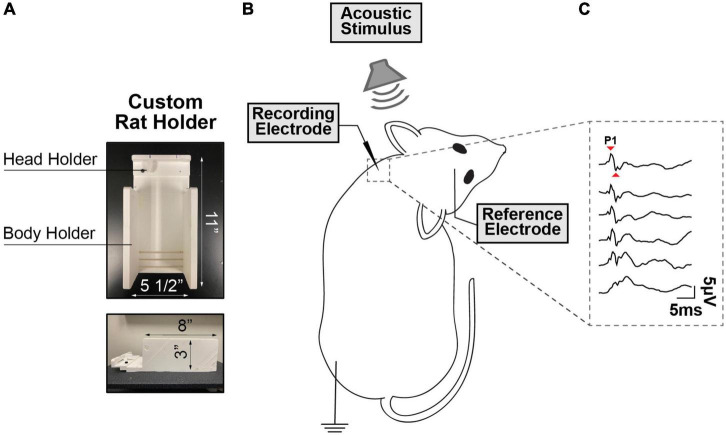
**(A)** Custom-Made Setup for cVEMP. The structure presents a body holder to keep the animal in place and a head holder to maintain the tension in the sternocleidomastoid muscle (SCM) **(B)** Rodent’s Placement. The rodent’s head is tilted in the opposite direction of the source of the acoustic stimulus and a recording electrode is placed perpendicularly through the sternocleidomastoid muscle. **(C)** Cervical Vestibular Evoked Myogenic Potentials (cVEMP). Example of cVEMP responses evoked by acoustic stimuli delivered via a speaker placed inside the left external auditory canal. (1 kHz, 100 to 50 dB SPL).

During vestibular testing, the animals were restrained using an injection cone whose end was cut to expose the head and neck, and ultimately secured on a custom-made platform (Figure 1A). The myogenic potentials were recorded from sternocleidomastoid muscle (SCM) (Kitamura and Sakai, 1982). To maintain proper muscle tension, a head holder anchored to the stage was used to turn and keep the rat’s head in place on one side at ∼90° angle. The head holder and stage were designed in SolidWorks and 3D printed at the University of Miami and are customizable for different sized rodents. A recording electrode (hypodermic needle EMG electrode) was inserted perpendicularly into the neck extensor muscle ipsilateral to the stimulated ear, the ground electrode was placed subcutaneously into the hindlimb, and the reference electrode was placed into the scalp at the vertex (Figure 1B). The ipsilateral SCM was selected as prior literature has shown that it is strongly recruited by ACS via a saccular-dominated response (Colebatch et al., 1994). A SmartEP system (Intelligent Hearing Systems, USA) was used to record VEMPs evoked by pure tone bursts from 1 to 16 kHz delivered via the ER3A earphone insert in the rodent’s external auditory canal. The myogenic potentials were collected at decreasing stimulus intensity, from 100 dB to 50 dB SPL following 10 dB steps. The signals were amplified by 100k and band-passed (30–1,000 Hz). Each recording was averaged from 256 sweeps, an acquisition rate = 5/s with alternating phase, and a sampling rate of 200 μs. cVEMPs were characterized by the presence of a biphasic waveform (positive P1 to negative N1) at 3–5 ms from the stimulus onset. Two consecutive traces were collected at any intensity level tested to verify the repeatability and stability of the signal. At every frequency, cVEMP thresholds were visually determined by blind investigation utilizing a recognizable P1-N1 peak as minimum criteria. The amplitude and latency of the P1 wave at a suprathreshold level of 90 dB SPL were also evaluated during these assessments (Figure 1C). To control for the influence of tonic muscle activity level on the cVEMP, in every experiment performed, continuous EMG signals of the rodents’ sternocleidomastoid (SCM) muscle were recorded over the 100 ms preceding each cVEMP stimulus onset. For every trace collected, the data acquisition system (Intelligent Hearing Systems, USA) provided the root mean square (RMS) as reliable estimators reflecting the average amplitude of the SCM’s EMG signals for the pre-stimulus interval considered.

The cVEMP measurements were carried out first on day 0 (labeled T_0_ or baseline) at three time points: 0 h, 2 h, and 4 h. The measurements were then repeated once each at day 1 (D1), day 3 (D3), and day 7 (D7).

### 2.3. Auditory testing (ABR)

Auditory brainstem responses (ABR) were measured on the same animals to determine the baseline hearing and assess safety of the cVEMP protocol used in this study. ABRs were performed before the initial vestibular assessment (T_0_) to establish baseline values and at D1, D3, and D7 prior to the corresponding cVEMP measurements. This allowed us to determine if the loud sounds presented during previous cVEMP assessments caused any observable changes in auditory thresholds. Pure-tone stimuli at 2, 4, 8, 16, 24, and 32 kHz and for a click stimulus of 1,000 μs (80 dB to 10 dB SPL, 10 dB intensity step) were delivered via in-ear speakers placed within auditory ear canals. ABRs were measured bilaterally as with cVEMPs. In a standard configuration, subcutaneous recording electrodes were placed behind each ear, with reference electrode at the skull vertex and a grounding electrode above the muscle in the hind leg. The subcutaneous needle electrodes were attached to a pre-amplifier and the IHS data acquisition system (Intelligent Hearing Systems, USA). System settings and protocol were previously described (Tamames et al., 2016), with calibration of the high- and low-frequency transducers performed for all tested intensities and frequencies. In this study, a recognizable Wave I peak was used as the minimum criteria to determine ABR thresholds.

### 2.4. Statistical analysis

Selection of individual statistical method depended on the type and distribution of the data obtained and nature of the observations. All the data presented have been analyzed using Prism (version 9.5.1, by GraphPad Software, Boston, MA). In every animal, the Interaural Asymmetry Ratios [IAR = (| P1-N1 X_Left_ – P1-N1 X_Right_) | /(P1-N1 X_Left_ + P1-N1 X_Right_) *100] were used to investigate possible right–left differences in cVEMP threshold, amplitude, and latency (IAR_Cutoff_: Amplitude ≤ 15% Latency ≤ 5% Threshold ≤ 5%). If absent, the values for all response parameters from both ears were averaged. The variability, normal distribution, and homogeneity of variance of the datasets presented were assessed using the Coefficient of Variation (CV%), Shapiro-Wilk, and Brown-Forsythe or Bartlett’s Tests, respectively. The statistical analysis performed includes One- and Two-Way ANOVA (*post-hoc* Tukey’s multiple comparison Test), Kruskal–Wallis Test (*post-hoc* Dunn’s multiple comparison Test), multiple paired, and unpaired *T*-Test (all of them corrected for multiple comparisons using the Holm-Sidak method). Test-retest reliability of baseline-adjusted longitudinal datasets was assessed via Repeated Measures One-Way ANOVA or Mixed-effect model (REML) (*post-hoc* Tukey’s multiple comparison Test). The repeatability of cVEMPs acquired at unequal time intervals between assessments was also estimated using the Intraclass Correlation Coefficients. ICCs are calculated using a Two-Way mixed effect (absolute agreement) single measurement model (Test-retest Reliability → Perfect: ICC = 1, Excellent: ICC ≥ 0.75, Fair-to-Good: 0.40 ≤ ICC < 0.75, Poor: ICC < 0.40) (Versino et al., 2001; Maes et al., 2009; Nguyen et al., 2010; Koo and Li, 2016). To evaluate data distribution between sex groups and/or frequencies, non-linear fits were compared using the Extra sum-of-squares F Test.

Unless stated, all the data are presented as absolute values (mean ± S.E.M) with following labels for statistical significance: *p < 0.05, ^**^p < 0.01, ^***^p < 0.001; ns = not statistically significant.

## 3. Results

### 3.1. Frequency-dependent variability in ACS-evoked cVEMPs

**FIGURE 2:**
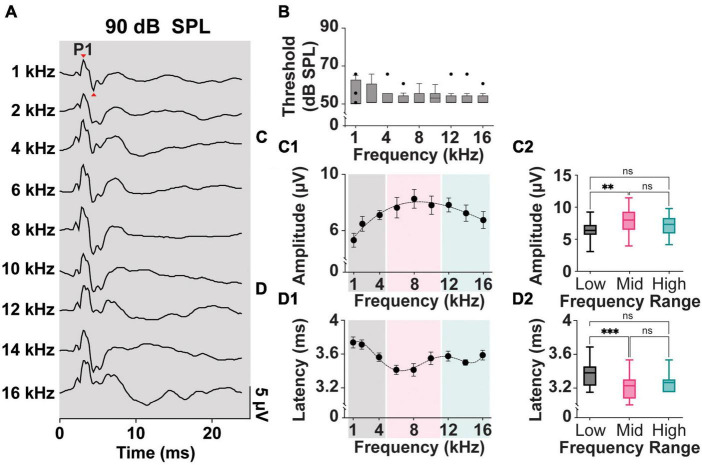
Threshold, Amplitude, and Latency comparison of Frequency-matched cVEMPs. **(A)** Example of cVEMP traces at a suprathreshold level of 90 dB SPL evoked by pure tone bursts from 1 to 16 kHz. **(B)** Vestibular thresholds measured across all the frequencies tested (*n* = 8). **(C1)** Cubic fit distribution of cVEMPs’ P1-N1 amplitudes at 90 dB SPL across frequencies (*R*^2^ = 0.96) (1–16 kHz). **(D1)** Fifth-order polynomial fit distribution of cVEMPs’ P1 latencies at 90 dB SPL across frequencies (*R*^2^ = 0.98) (1–16 kHz). **(C2)** Amplitude and **(D2)** latency of the responses were grouped based on the stimulus tone (Low: 1–4 kHz; Mid: 6–10 kHz; High: 12–16 kHz) and compared (mean ± S.E.M). ^**^p < 0.01 and ^***^p < 0.001. ns = not significant.

Figure 2A shows typical ipsilateral cVEMPs elicited by ACS delivered at 90 dB SPL at frequencies from 1 to 16 kHz (100% response rate). In human subjects the positive and negative peaks appear at 13 and 23 ms, respectively (named p13 and n23 peaks). In rodent species, including rats, guinea pigs, and mice, the positive (P1)-negative (N1) cVEMP waveforms recorded from the neck extensor muscle have been reported between 3–9 ms (Yang et al., 2010b; Zhu et al., 2011; Curthoys, 2017; Negishi-Oshino et al., 2019).

In our setup, we could reliably and repeatedly evoke clearly identifiable P1 and N1 waveforms in a rat model across multiple frequencies and at different stimulus intensities. Figure 2B shows a comparison of the cVEMP thresholds, as measured by the intensity of ACS evoking clearly identifiable P1-N1, across the frequencies tested. Kruskal–Wallis Test and *post-hoc* Dunn Test (*p < 0.05) indicate that this parameter is not significantly affected by the stimulus tone. Across the rats and independent of sex, 50 dB SPL was the lowest intensity input able to evoke a myogenic potential (threshold) for all frequencies tested.

Figures 2C, D, show the mean P1-N1 amplitudes and P1 latencies of cVEMPs elicited at suprathreshold 90 dB SPL across frequencies tested from 1 to 16 kHz. A third-order polynomial equation fits the distribution of the amplitudes with a significant peak at 8 kHz (*R*^2^ = 0.96, Figure 2C1). Similarly, the fifth-order polynomial equation fitting the latency values (*R*^2^ = 0.98) presents a significant negative peak around the same frequency (8 kHz, Figure 2D1). To reveal possible frequency effect on cVEMP responses, we grouped and averaged the data for frequencies between 1–4 kHz (Low frequency), 6–10 kHz (Mid frequency), and 12–16 kHz (High frequency). Figure 2C2 shows that the amplitude of the cVEMPs evoked by stimuli in the mid-frequency range were significantly larger than amplitudes evoked by low frequencies (7.9 ± 0.3 μV, One-way ANOVA and *post-hoc* Tukey Test, *p < 0.05). Similarly, Figure 2D2 highlights that cVEMP responses elicited by mid-frequency stimuli present at significantly shorter latencies compared to the myogenic potentials evoked by stimuli between 1 and 4 kHz (3.4 ± 0.04 ms, Kruskal-Wallis Test and *post-hoc* Dunn Test, *p < 0.05; Figure 2D2). While there was a trend, neither the amplitude nor latency of the response evoked by mid-frequency stimuli were statistically different compared to those evoked by high-frequency stimuli.

Mean P1-N1 amplitudes and P1 latencies measured at 90 dB SPL for all the frequency ranges presented in this study are further listed in Table 1 (*Mixed Cohort, n* = 8) and are also separated by sex (*n* = 4, male and female each).

**TABLE 1:** Mean P1-N1 amplitudes and P1 Latencies.

Frequency range (kHz)	cVEMP parameter					
	Amplitude (μ V)			Latency (ms)		
	Mixed cohort	Females	Males	Mixed cohort	Females	Males
1–4	6.31 ± 0.30	6.56 ± 0.45	6.04 ± 0.52	3.67 ± 0.03	3.71 ± 0.05	3.61 ± 0.04
6–10	7.90 ± 0.38	8.34 ± 0.59	8.02 ± 0.49	3.46 ± 0.04	3.55 ± 0.05	3.31 ± 0.03**
12–16	7.27 ± 0.32	7.75 ± 0.41	7.62 ± 0.46	3.55 ± 0.03	3.61 ± 0.04	3.47 ± 0.02*

cVEMP amplitude and latency are grouped based on the stimulus tone. Data are expressed as mean ± S.E.M. Both response parameters are compared between male and female cohort within the respective frequency range. *p < 0.05, **p < 0.01.

### 3.2. Reliability of SCM muscle activity

**FIGURE 3:**
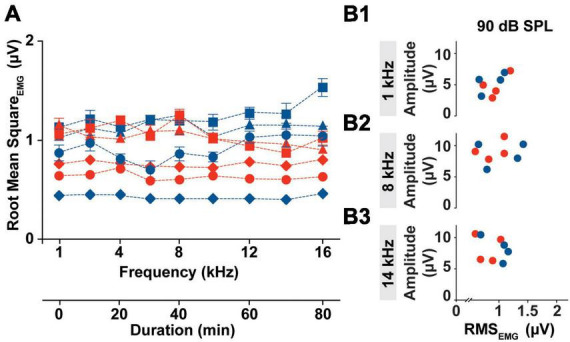
Intra-subject Variability of the SCM muscle’s tonic activity. **(A)** Averaged RMS values of EMG amplitudes collected from all the subject (Females → red symbol; Males → blue symbol) prior the specific stimulus onset (1–16 kHz) during an individual cVEMP testing session (T0 hr). **(B1–B3)** Bivariate plots to test correlation between P1 amplitude elicited by 90 dB SPL stimulation at 1, 8, and 14 kHz and the Root Mean Square of the EMG amplitude measured over the pre-stimulus interval of the correspondent trace (100 ms).

Although cVEMP amplitude measures have become the most useful tool for the detection of abnormality in clinical populations, it presents high level of inter- and intra- subject variability. One of the challenges with the reliability of myogenic potentials recorded from the neck extensor, is the influence of the muscle tonic activity on cVEMP amplitude. In Figure 3, we evaluated the tonic level of the SCM muscle within the experimental timeframe for all animals.

In the current setup with the acquisition protocol used (including the number of responses recorded, the rate of acquisition, and the repeated sweeps per trace), the time required to evoke cVEMP traces unilaterally for each frequency was ∼10 min. Overall, the cVEMP recording session lasted approximately 90 min for every ear. As highlighted in Figure 3A, the root mean squared (RMS) activity of the EMG measured over a 100 ms period prior to stimulus onset, did not vary significantly over the duration of the experiment (CV_AVG_% < 6, Mixed-effects model with Geisser-Greenhouse correction and *post-hoc* Tukey Test, *p < 0.05), indicating low intra-animal variability. The averaged RMS values measured did reveal an inter-animal variability with a non-significant sex effect (Mixed-effects model with Geisser-Greenhouse correction and *post-hoc* Tukey Test, *p < 0.05). Notably, the differences in the tonic activity of the sternocleidomastoid muscle observed between rats were not associated with significant changes in the amplitude of the corresponding cVEMP at any of the frequency tested (1 to 16 kHz). In the examples provided in Figure 3B, we show that there is no correlation between the P1 amplitude of the vestibular response elicited by 90 dB SPL at 1, 8, or 14 kHz and the RMS value measured from the 100 ms preceding the correspondent stimulus onset.

Root mean square measured from every subject are averaged and grouped based on the stimulus tone used following EMG assessment. The 95% confidence intervals for each subset are listed in Supplementary Table 1.

### 3.3. Intensity-dependent variability of ACS-evoked cVEMPs

**FIGURE 4:**
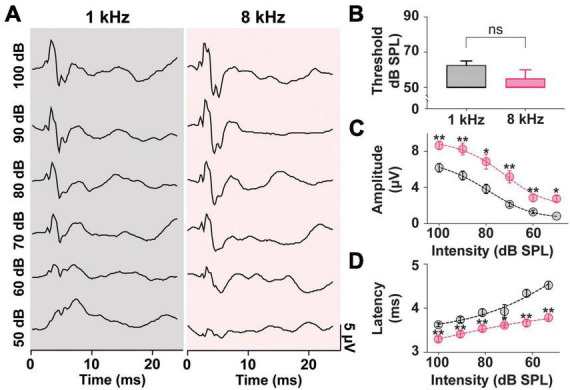
Threshold, Amplitude, and Latency comparison of Intensity-matched cVEMPs evoked at 1 and 8 kHz. **(A)** cVEMP responses to stimulation at 1 and 8 kHz (100 to 50 dB SPL). **(B)** Comparison of cVEMP thresholds observed at 1 and 8 kHz. Non-linear fits of intensity-dependent distribution of **(C)** cVEMPs wave I (P1-N1) amplitudes and **(D)** latencies at 1 and 8 kHz. *p < 0.05, ^**^p < 0.01, and ns = not significant.

In prior literature, the frequency tuning of the saccule highlighted preferred frequencies of 500–1,000 Hz (Young et al., 1977). As such ACS tone bursts around these frequencies have been the preferred stimuli in both clinical and preclinical studies (Colebatch et al., 2016; Corneil and Camp, 2018; Negishi-Oshino et al., 2019; Rosengren et al., 2019). As seen in Figure 2, however, in a rat model, stimuli at 8 kHz resulted in robust, reliable, and largest cVEMPs. To further study the effects of ACS intensity and frequency on cVEMP responses, a detailed analysis of thresholds, amplitudes, and latencies of cervical myogenic potentials evoked by pure tone burst stimuli at 1 and 8 kHz were performed (Figure 4). There was no significant difference between the cVEMP thresholds for either 1 kHz or 8 kHz ACS (Figure 4B, Wilcoxon matched-pairs signed rank test, *p < 0.05). We could reliably evoke cVEMPs at 50 dB SPL. However, we did observe differences in amplitudes and latencies at these frequencies. In Figure 4C, the distributions of the amplitudes of the responses evoked by both 1 and 8 kHz stimuli at intensity from 100 dB to 50 dB SPL (10-dB steps) are shown along with fit dose-response curves. While there was no significant difference between the hillslope at 1 and 8 kHz (1 kHz:9.17; 8 kHz:10.47; Extra sum-of-squares F Test, *p < 0.05), the P1 amplitude of cVEMPs evoked by 8 kHz stimuli at every intensity tested are, on average, larger by 2.53 ± 0.24 μV compared to the amplitude of responses evoked by 1 kHz tone bursts at the same intensity stimuli (Multiple Paired *T*-Test corrected for multiple comparisons with Holm-Sidak method, *p < 0.05). Figure 4D presents the distributions of the latencies of the vestibular responses evoked by both 1 and 8 kHz stimuli across intensities tested. Again, the dose-response curves reveal no significant difference between hillslopes (1 kHz: −5.63; 8 kHz: −3.31; Extra sum-of-squares F Test). However, there was an overall difference between latencies of responses at 1 vs. 8 kHz. At each intensity, stimulation at 8 kHz evoked a response at a shorter latency (average shift: −0.47 ± 0.1 ms) when compared with the same intensity stimuli at 1 kHz (Multiple Paired *T*-Test corrected for multiple comparisons with Holm-Sidak method, *p < 0.05).

### 3.4. Sex-related differences in cVEMPs

**FIGURE 5:**
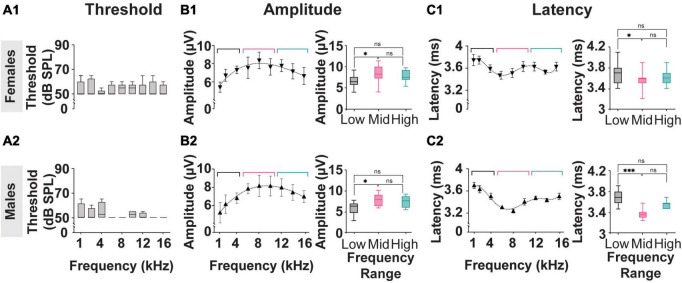
Threshold, Amplitude, and Latency comparison of Frequency-matched cVEMP responses in Male and Female rats. **(A1,A2)** Vestibular thresholds measured across all the frequencies tested in female and male subjects. The box plots represent the mean and interquartile ranges of the threshold values, and the error bars above and below the box indicate the max and min values observed, respectively. **(B1,B2)** Cubic distribution of amplitude data in both groups (Females: *R*^2^ = 0.91 Males: *R*^2^ = 0.98) **(C1,C2)** Fifth order polynomial distribution of latency data from male and female subjects (Females: *R*^2^ = 0.99 Males: *R*^2^ = 0.95). Inserts present comparisons of the correspondent data grouped according to the stimulus tone used (Low: 1–4 kHz; Mid: 6–8 kHz; High: 12–16 kHz). *p < 0.05, ^***^p < 0.001, and ns = not significant.

The results described in Figure 2 reflect analysis performed on averaged data acquired from both female and male subjects used in this study. As shown in Figure 5, the threshold, amplitude, and latency of the myogenic potentials evoked in females (*n* = 4) and males (*n* = 4) from the *Mixed Cohort* are analyzed separately and compared. The P1-N1 amplitudes and P1 latencies marginal means are listed in Table 1.

Consistent with the data presented in Figures 2B, 5A1, A2 shows that, within each sex group, the thresholds of the cVEMP response do not vary significantly across the frequency range (1–16 kHz) (Kruskal–Wallis Test and *post-hoc* Dunn Test, *p < 0.05). No significant difference is observed when comparing the frequency-matched amplitudes between females and males (Mixed-effects model with Geisser-Greenhouse correction and *post-hoc* Tukey Test, *p < 0.05).

Figures 5B, C present the amplitude and latency values of cVEMP responses to stimuli between 1 and 16 kHz at 90 dB SPL. Consistent with the data shown in Figures 2C, D, the distributions of amplitude and latency data fit third- and fifth-order polynomial equations, respectively for both the female and male subgroups. Averaged amplitude values for the low-frequency (1–4 kHz), mid-frequency (6–10 kHz), and high-frequency (12–16 kHz) ranges are not significantly different between male and female subjects (Table 1, Females vs Males; Two-Way ANOVA and *post-hoc* Tukey Test, *p < 0.05). Figures 5B1, B2 *inserts* show that stimuli in the mid-frequency range evoke responses significantly larger only than the ones obtained by lower frequencies (Table 1, Females vs Males; Two-Way ANOVA and *post-hoc* Tukey Test, *p < 0.05). The analysis of the latency of cVEMPs from female rats (Figure 5C1 *insert*) shows that vestibular responses evoked by stimuli between 6–10 kHz are significantly faster than the ones evoked by stimulation in the low-frequency range (1–4 kHz) but not significantly different from those evoked by 12–16 kHz inputs (Table 1, Mixed-effects model with Geisser-Greenhouse correction and *post-hoc* Tukey Test, *p < 0.05). Similarly, cVEMPs evoked by mid-frequency stimulations in male subjects are significantly faster only than the ones evoked by low-frequency range stimuli (Figure 5C2 *insert*, Mixed-effects model with Geisser-Greenhouse correction and *post-hoc* Tukey Test, *p < 0.05). Latency comparisons between female and male subjects reveal significant sex effects on cVEMPs evoked by 6–10 and 12–16 kHz stimuli (Table 1, Mixed-effects model with Geisser-Greenhouse correction and *post-hoc* Tukey Test, *p < 0.05).

### 3.5. Reliability and replicability of cVEMPs

**FIGURE 6:**
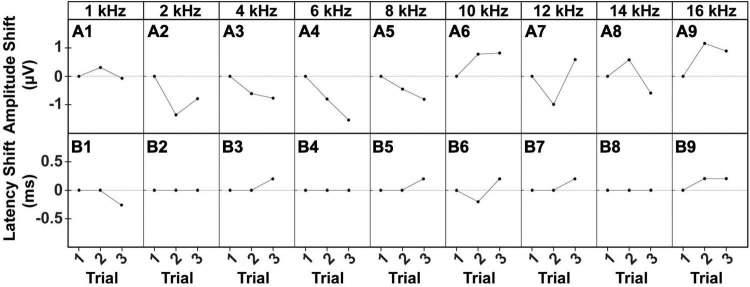
Intra-subject Variability of ACS-evoked cVEMPs. Example of **(A1–A9)** amplitude and **(B1–B9)** latency shifts of two consecutive cVEMPs (Trial #2, Trial #3) from the correspondent baseline value (Trial #1) evoked by stimuli at 90 dB SPL from 1 to 16 kHz in a male subject.

To assess reliability and replicability of the cVEMP response during an experimental session (same day), we repeated the measurements in duplicates or triplicates. Figure 6 shows the baseline-corrected P1-N1 amplitudes and P1 latencies evoked in a single representative animal over three consecutive cVEMP response acquisitions (Trial #1, #2, and #3) at T_0_. As with all prior experiments, cVEMPs were repeatably elicited in response to ACS at 100 to 50 dB SPL from 1–16 kHz. The plots A1-A9 and B1-B9 represent repeated measures occurring at 90 dB SPL and show the same setup as follows. The respective amplitudes and latencies measured from the first cVEMP evoked at each frequency is set as baseline (Trial #1, horizontal dotted line at 0). Amplitude and latency shift from the baseline are then quantified for the subsequent trials (Trial #2 and #3). Statistical analysis using repeated measures One-Way ANOVA or a Mixed-effect model with Geisser-Greenhouse correction showed that, across all the frequencies, both the cVEMP metrics do not vary significantly between consecutive measurements (*p < 0.05). These findings indicate good intra-subject reliability of the vestibular responses evoked. Table 2 shows the 95% confidence intervals of the raw cVEMP metrics measured from every animal in the mixed cohort at 90 dB SPL at each frequency. The coefficients of variation associated indicate the inter-subject variability for every frequency-specific threshold, amplitude, and latency dataset. Non-normalized P1 latency and threshold had lower coefficients of variation than the frequency-matched P1-N1 amplitude. For all the response parameters, the frequency effect on the coefficients was non-significant (Kruskal-Wallis Test and *post-hoc* Dunn Test, *p < 0.05).

**TABLE 2:** Inter-subject variability of ACS-evoked cVEMPs.

Frequency (kHz)	cVEMP parameter								
	Threshold (dB SPL)			Amplitude (μ V)			Latency (ms)		
	95% confidence interval of mean^A^		CV%^B^	95% confidence interval of mean^A^		CV%^B^	95% confidence interval of mean^A^		CV%^B^
	Lower bound	Upper bound		Lower bound	Upper bound		Lower bound	Upper bound	
1	48.7	60.0	4.37	4.15	6.50	23.61	3.58	3.89	4.2
2	49.2	59.6	4.37	5.25	7.72	11.75	3.58	3.84	3.32
4	48.7	57.6	7.37	6.34	7.88	10.73	3.44	3.68	3.38
6	48.8	55.0	4.63	5.92	9.35	16.68	3.28	3.54	3.60
8	49.3	55.7	3.25	6.68	9.84	10.46	3.24	3.59	2.50
10	50.0	56.2	4.87	6.27	9.35	16.55	3.38	3.72	2.91
12	48.0	57.0	3.00	6.65	9.01	19	3.44	3.71	3.84
14	48.0	57.0	3.00	5.87	8.61	16.59	3.42	3.58	3.96
16	48.8	55.0	1.625	5.34	8.17	15.24	3.45	3.72	2.41

Within the cohort studied (*n* = 8), cVEMP threshold and amplitude/latency datasets of responses evoked by stimuli at 90 dB SPL from 1 to 16 kHz are described by the **(A)** 95% Confidence Interval (95% CI) and the correspondent (B) Coefficient of Variation. (Low: CV < 10% Medium: 10% < CV < 20% High: CV > 20%. Very High: CV > 30%).

**FIGURE 7:**
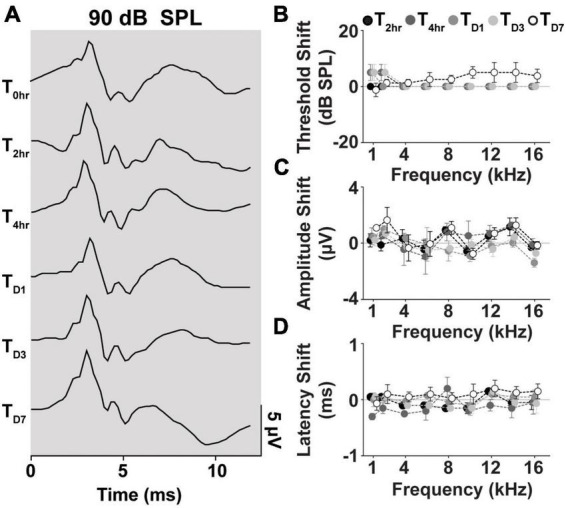
Longitudinal comparison of Threshold, Amplitude, and Latency of Frequency-matched cVEMPs. **(A)** Example of cVEMP traces at a suprathreshold level of 90 dB SPL evoked by 1 kHz pure tone bursts at different time points (T → 0 h, 2 h, 4 h, D1, D3, D7). Comparison of **(B)** threshold, **(C)** amplitude, and **(D)** latency shift (mean ± S.E.M) from the initial cVEMP assessment for every timepoint considered in this study at every frequency tested (1–16 kHz).

We further evaluated cVEMP replicability over several time points in a subset of the cohort studied (*n* = 2 male and female each). Figure 7A shows the replicability of the waveform across each time point. Figures 7B–D shows the total cVEMP threshold, amplitude, and latency shifts between experimental sessions occurring at 2 h, 4 h, D1, D3, and D7 from the initial vestibular assessment (T_0hr_ = baseline). For every response parameter, no significant difference was observed between repeated measures for any of the frequencies (Mixed-effect model and *post-hoc* Tukey Test, *p < 0.05). Similarly, the Intraclass Correlation Coefficients calculated for the raw longitudinal datasets indicate Good-to-Excellent Test-retest reliability of the cVEMP metrics acquired over time (ICC_Threshold_ > 0.75; ICC_Amplitude_ > 0.75; ICC_Latency_ > 0.60).

### 3.6. SCM muscle activity across days

**FIGURE 8:**
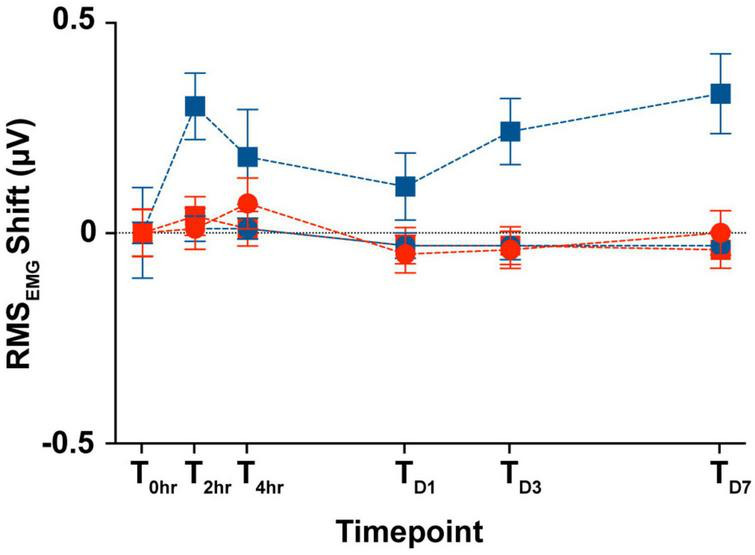
Longitudinal comparison of the SCM muscle’s tonic activity. Comparison of the averaged RMS_EMG_ shift (mean ± S.E.M) from the initial cVEMP assessment for every experimental session considered in this study (T → 0 h, 2 h, 4 h, D1, D3, D7).

In the animals from the longitudinal subgroup, we compared the SCM tonic activity across the six time points as a proxy for custom-designed cVEMP setup used to perform all the experiments in limiting any variability introduced by the operator. Figure 8 shows the changes between the RMS values (see Section “2. Materials and methods”) measured during the first cVEMP recording session (T_0hr_ = baseline) and the ones acquired over the subsequent time points. A Mixed-effect model analysis (*post-hoc* Tukey Test, *p < 0.05) and the correspondent Intraclass Correlation Coefficients reveal, respectively no significant time, sex effects, and excellent Test-retest reliability of the RMS values acquired (ICC_RMS_ > 0.8).

## 4. Discussion

Despite the wide clinical utility of VEMPs to characterize otolithic function in humans, there is no established pre-clinical model for the ocular or cervical vestibular myogenic responses. The few published studies testing cVEMPs in rodents offer setups or protocols that are not standard and/or lead to significant variability in the response rate and their amplitudes or latencies across groups. The present study successfully established a preclinical rodent model with detailed characterization of sound-evoked cervical myogenic potentials. The cVEMP and its associated parameters, such as threshold, latency, and amplitude, make feasible testing for the integrity of sacculocollic pathway using a non-invasive method. While several preclinical and clinical studies have focused on detailing the neural basis and the functional anatomy underlying these vestibular evoked responses (Murofushi et al., 1995; Nazareth and Jones, 1998; Curthoys et al., 2011; Zhu et al., 2011, 2014), only a few studies have focused on characterization of cVEMPs to test vestibular function in animal models presenting either intact labyrinth (Yang and Young, 2005; Yang et al., 2010a; Zhu et al., 2011; Ya et al., 2016) or pathological conditions (Shojaku et al., 2007; Sheykholeslami et al., 2009; Yang et al., 2010b). In these works, the lack of a standardized protocol and the use of a set up that is not easily adaptable to a small rodent model introduces variability and inconsistency across studies, making it difficult to compare and replicate findings.

In our setup, with the animals anesthetized, the positioning and orientation of the head and body in a simple 3D printed holder allow for uniform tension on the neck extensor muscles on either side (Figure 3). Cervical myogenic potentials were evoked via air-conducted sounds (ACS), given that they are the preferred stimuli in clinical settings. Comparable vestibular responses to ACS were evoked both in female and male rats and were consistent and repeatable in every subject over many hours and several experimental sessions, without any acute deleterious effects on the peripheral auditory system (Supplementary Figure 2). The peak frequency of cVEMP tuning curves was not affected by sternocleidomastoid muscle’s tonic level or the stimulus intensity (Supplementary Figure 1).

For the first time, in a preclinical model, the present study characterizes cVEMP responses to sounds at multiple frequencies. Interestingly, the sacculocollic VEMP responses in the rat showed a stimulus frequency tuning. The saccule, one of the two otolithic organs in the vestibular system, primarily functions in detecting linear acceleration and head tilts. While the primary role of the saccule is to sense gravity and linear movements (Fernandez and Goldberg, 1976), it has also been observed to exhibit responses to specific sound stimuli (Young et al., 1977; Cazals et al., 1983; Didier and Cazals, 1989), particularly at high frequencies (Curthoys et al., 2016). Our observations reveal an increased amplitude and reduced onset latency of the response with increasing frequency, with the peak response occurring between 6–10 kHz. Several previous clinical studies (Donnellan et al., 2010; Lewis et al., 2010; Wei et al., 2013) have shown that vestibular evoked myogenic potentials have a higher tuning peak for air-conduction compared to bone-conducted stimuli (∼1 kHz vs. 0.4 kHz). The otoliths, particularly the utricle, have been found to respond to frequencies up to several kilohertz (Curthoys et al., 2016). Recordings from utricular primary afferent neurons, often characterized by irregular firing rates, indicate that responses can be synchronized up to 2–3 kHz. This synchrony holds true for both bone-conducted vibration and air-conducted sound stimuli. While bone-conducted vibration stimuli appear to be more effective and reliable at lower frequencies, even responses to 3 kHz sound stimuli exhibit relatively low thresholds (Curthoys et al., 2011, 2012; Zhu et al., 2011). Similarly, measurements from toadfish primary afferents show high phase-locking up to 1 kHz (Highstein et al., 1996). Curthoys et al. (2006), Curthoys et al. (2017), and Curthoys et al. (2018) have conducted further investigations into utricle mechanics, fluid dynamics, and the neuroepithelial layer to address the neural basis for phase locking in utricular afferents in response to high-frequency stimuli. Studies by Pastras et al. (2017, 2023) recording utricular vestibular microphonics and on the mathematical model of excitation of the utricular macula, provide additional support to these results, showing that air-conducted stimuli can indeed produce responses in mammalian receptors at such high frequencies. However, what remains uncertain is whether otolith receptors exhibit tight phase locking sufficient to evoke a robust muscular response, as observed here at 8 kHz. It is possible that the location of the saccule and its mechanical coupling with the cochlea and the basilar membrane further renders it sensitive to air-conducted stimuli. Considering the complexity of receptor subtypes and macular geometry, coupled with the current findings, further research is necessary to comprehensively elucidate the intricate mechanisms underlying the tuning of the saccule’s response to sound stimuli at several kilohertz.

The results in this study suggest that cVEMPs and associated clinically relevant metrics can provide a reliable and repeatable non-invasive diagnostic test in a preclinical setting with significant implications for understanding the pathophysiology of vestibular disorders and assessing potential efficacious therapies. By utilizing the established model and the frequency tuning phenomenon observed in VEMP responses, researchers can investigate how specific vestibular disorders affect the responsiveness of the vestibular system at different frequencies. For instance, in conditions such as Meniere’s disease, characterized by endolymphatic hydrops, the abnormal fluid dynamics may lead to alterations in frequency tuning and cVEMP responses (Maheu et al., 2017; Murofushi et al., 2017; Angeli and Goncalves, 2019). Semicircular canal dehiscence may also induce a similar shift in frequency sensitivity (Songer and Rosowski, 2005; Curthoys and Grant, 2015; Iversen et al., 2018). As seen in our companion study investigating noise-induced vestibular deficits in firefighters, as well as studies from preclinical models of noise exposure, cVEMPs can be particularly useful in detecting early and long-term changes and potentially identifying the neural basis for the observed deficits. Further, the ability to reliably elicit cVEMP responses using clinically relevant-ACS in an animal model allows for forward and backward translation where pertinent questions underlying important clinical findings can be examined. This model presents novel opportunities and expands the scientific scope for assessing the safety and efficacy of different therapeutic approaches.

In conclusion, these findings provide evidence of the value and effectiveness of using VEMPs for diagnostic purposes in an animal model of vestibular disorders. The observed stimulus frequency tuning offers new insights into the vestibular evoked myogenic responses and opens avenues for further investigations of their underlying neural mechanisms.

## Data availability statement

The raw data supporting the conclusions of this article will be made available by the authors, without undue reservation.

## Ethics statement

The animal study was approved by the Institutional Animal Care and Use Committee, University of Miami. The study was conducted in accordance with the local legislation and institutional requirements.

## Author contributions

FR, YM, HS, and SR contributed to the conception and design of the study. FR and YM collected the data. FR and SR analyzed the data. FR performed all the statistical analysis. All authors contributed to the manuscript preparation, its revision, read, and approved the submitted version.
