# Supplementary material for: A reliable and reproducible protocol for sound-evoked vestibular myogenic potentials in rattus norvegicus
Source: Front Integr Neurosci. 2023 Sep 5;17:1236642. doi: 10.3389/fnint.2023.1236642 (PMC10508189; doi:10.3389/fnint.2023.1236642)
Supplement: Supplementary file 1 [file Image_1.pdf]

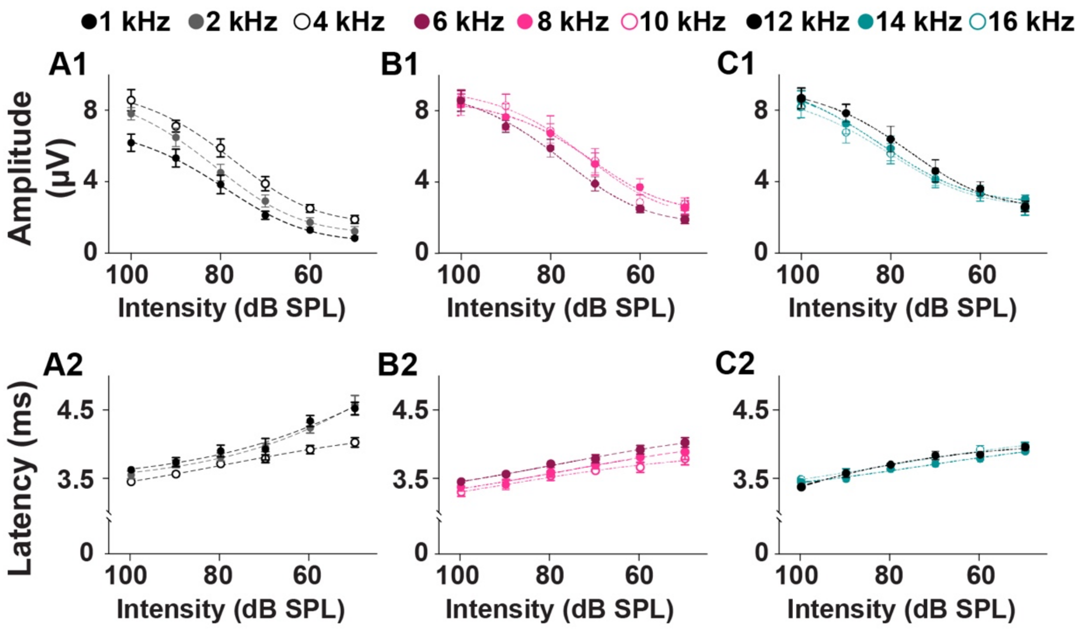

Figure S1. Intensity-dependent Variability in ACS-evoked cVEMPs. Dose-response curves (100 to 50 dB SPL) of wave I (P1-N1) **(A1-C1)** amplitudes and **(A2-C2)** latencies of cVEMP responses to stimuli at 1 to 4 kHz, 6 to 10 kHz, and 12 to 16 kHz. For each parameter considered, the independent non-linear fits were compared across frequencies with a global fit sharing Hillslope ($Hillsope_{Amplitude}$= 7.68; $Hillsope_{Latency}$= -3.31). No significant difference was observed between the two models. Hence, the Hillslope is the same for all datasets. Extra sum-of-squares F Test, *p<0.05.
