# Supplementary material for: A reliable and reproducible protocol for sound-evoked vestibular myogenic potentials in rattus norvegicus
Source: Front Integr Neurosci. 2023 Sep 5;17:1236642. doi: 10.3389/fnint.2023.1236642 (PMC10508189; doi:10.3389/fnint.2023.1236642)
Supplement: Supplementary file 2 [file Image_2.pdf]

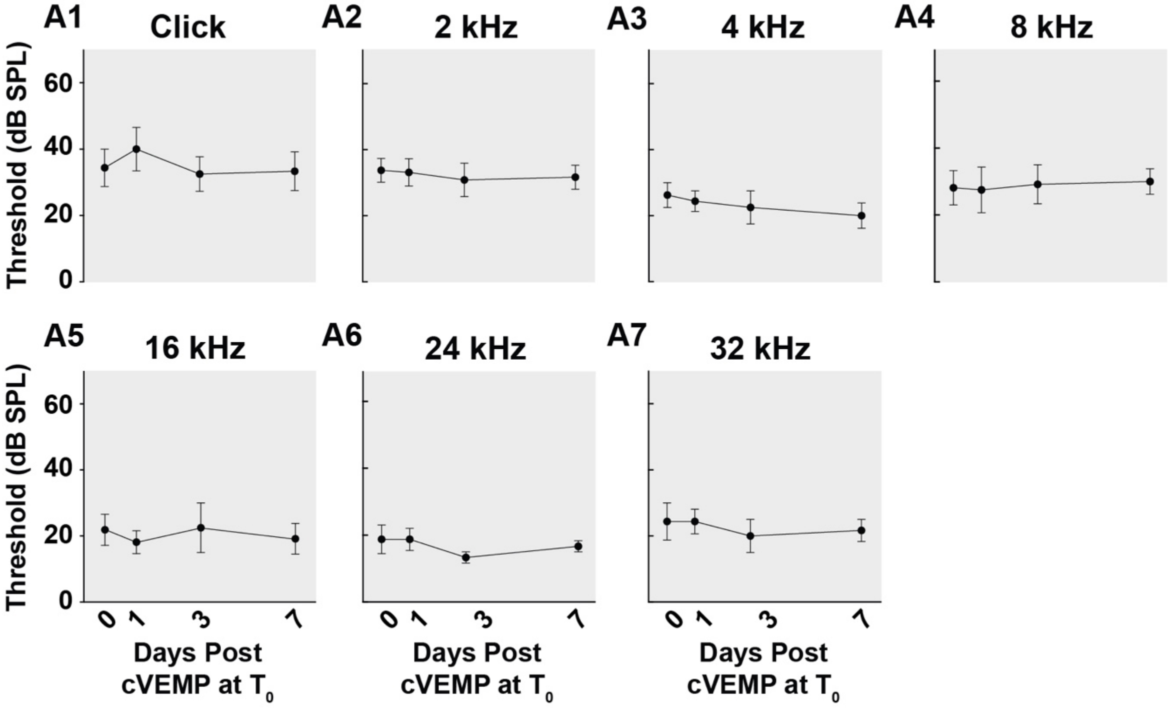

Figure S2. Safety of ACS cVEMP Protocol. (A1-A7) Auditory brain stem response (ABR) measurements of Brown Norway rats at different frequencies (click, 2, 4 8, 16, 24, 32 KHz) recorded before the initial vestibular test to establish baseline values (pre ABR=0) and at D1 D3, and D7 prior to the correspondent cVEMP measurement to determine if the repeated assessments caused any change in auditory thresholds compared to control (pre ABR). In all the frequencies tested, we show no significant shift in the ABR threshold at any of the timepoint considered. Statistical analysis included One-way ANOVA followed by post hoc Dunnett's test for Click, 2 kHz, 8 kHz, 16 kHz, 24 kHz, and 32 kHz. Because of the non-Gaussian distribution of the 4 kHz dataset, the Kruskal-Wallis test followed by post hoc Dunnett's was used. $*p<0.05$
