# Supplementary material for: A reliable and reproducible protocol for sound-evoked vestibular myogenic potentials in rattus norvegicus
Source: Front Integr Neurosci. 2023 Sep 5;17:1236642. doi: 10.3389/fnint.2023.1236642 (PMC10508189; doi:10.3389/fnint.2023.1236642)
Supplement: Supplementary file 3 [file Table_1.pdf]

**Frequency (kHz)**	**Root Mean Square EMG Amplitude (μV)**	
	**95% Confidence Interval of Mean[A]**	
	Lower Bound	*Upper Bound*
**1**	0.67	1.10
**2**	0.69	1.30
**4**	0.68	1.11
**6**	0.62	1.09
**8**	0.65	1.16
**10**	0.64	1.07
**12**	0.66	1.14
**14**	0.64	1.13
**16**	0.67	1.22

Table S1. 95% Confidence Interval of Mean of SCM tonic activity in the rat.
